# Astrocyte dysfunction in Parkinson's disease: from the perspectives of transmitted α-synuclein and genetic modulation

**DOI:** 10.1186/s40035-021-00265-y

**Published:** 2021-10-18

**Authors:** Changjing Wang, Tongtong Yang, Meiyu Liang, Junxia Xie, Ning Song

**Affiliations:** 1 Department of Physiology, Shandong Provincial Key Laboratory of Pathogenesis and Prevention of Neurological Disorders, School of Basic Medicine, Qingdao University, Qingdao, 266071 China; 2 Institute of Brain Science and Disease, Qingdao University, Qingdao, 266071 China

**Keywords:** Parkinson’s disease, Astrocytes, Genetic mutation, α-synuclein, Therapeutics

## Abstract

Parkinson’s disease (PD) is a common neurodegenerative disorder that primarily affects the elderly. While the etiology of PD is likely multifactorial with the involvement of genetic, environmental, aging and other factors, α-synuclein (α-syn) pathology is a pivotal mechanism underlying the development of PD. In recent years, astrocytes have attracted considerable attention in the field. Although astrocytes perform a variety of physiological functions in the brain, they are pivotal mediators of α-syn toxicity since they internalize α-syn released from damaged neurons, and this triggers an inflammatory response, protein degradation dysfunction, mitochondrial dysfunction and endoplasmic reticulum stress. Astrocytes are indispensable coordinators in the background of several genetic mutations, including *PARK7, GBA1, LRRK2, ATP13A2, PINK1, PRKN* and *PLA2G6*. As the most abundant glial cells in the brain, functional astrocytes can be replenished and even converted to functional neurons. In this review, we discuss astrocyte dysfunction in PD with an emphasis on α-syn toxicity and genetic modulation and conclude that astrocyte replenishment is a valuable therapeutic approach in PD.

## Background

Parkinson’s disease (PD) is the second-most common neurodegenerative disorder, affecting 2%–3% of the population over 65 years of age [1]. Although most PD cases are sporadic with unknown etiology, approximately 5%–10% of PD cases are familial, suggesting that genetic vulnerability is a risk factor for PD [2, 3]. The neuropathology of PD is primarily characterized by progressive degeneration of dopaminergic neurons in the substantia nigra pars compacta (SNpc) and the formation of Lewy bodies in the surviving neurons [1, 4]. Aggregated forms of α-synuclein (α-syn) are the major component of Lewy bodies, which are considered the pivotal mechanism underlying neurodegeneration that occurs in PD [5, 6]. Physiologically, α-syn is found mainly in neurons, functioning in membrane-associated processes at the presynaptic level. It interacts with the membrane to maintain synaptic vesicle trafficking, and serves as a molecular chaperon with interactions with target proteins. In dopaminergic neurons, α-syn is closely related to dopamine metabolism and the loss of functional α-syn might result in dysregulation of dopamine synthesis, transport and storage [7–10]. Lower expression of α-syn is consistently detected in astrocytes. ​Meanwhile, mice selectively overexpressing A53T α-syn in astrocytes show widespread astrogliosis, dopaminergic neuronal loss and movement disabilities, suggesting that the synthesized α-syn in astrocytes is sufficient to impair normal functions of astrocytes and initiate neurodegeneration [11]. A recent study has reported that astrocytes derived from induced pluripotent stem cells (iPSCs) of PD patients manifest α-syn aggregates and a state of higher activation, indicating that α-syn synthesized in astrocytes could be unfavorable when present excessively or under pathological conditions [12].

A number of studies have shown that toxic/aggregated species of α-syn such as oligomers, protofibrils and fibrils can be efficiently internalized by astrocytes, raising the idea that α-syn could be transported from neurons to astrocytes [13, 14]. This unusual property has led investigators to propose that α-syn possesses “prion-like” properties that allow it to spread pathology in the brain [15]. Several studies have indicated that astrocytes take up extracellular α-syn rapidly via endocytosis [13, 16–19]. In addition to the uptake from extracellular surroundings, the presence of tunneling nanotubes (TNTs) among cells suggests the existence of a previously unknown mechanism underlying the transfer of α-syn from astrocyte to astrocyte. Human astrocytes derived from embryonic stem cells (ESCs) have been shown to actively transfer aggregated α-syn to healthy astrocytes via TNTs [20]. Finally, exosomes are considered important mediators of α-syn transmission. Astrocytes are able to secrete and receive exosomes from other cell sources [21], and the exosome-mediated removal of aggregated α-syn species could be achieved via internalization of exosomes by astrocytes [22, 23].

As the most abundant glial cells constituting 20% to 40% of all neuroglia in various brain regions [24], astrocytes display a broad range of functions that are indispensable for normal development and function of the brain. These functions include providing structural support for neural networks and providing nutrients and energy for neurotrophic support, maintenance of water hemostasis through regulation of aquaporins, maintenance of the extracellular ion balance, and repair of damage within the brain [25, 26]. More recently, the presence of abundant perisynaptic astrocytic processes has led to investigations of novel roles of astrocytes in the formation and function of synapses [27–29]**.** Astrocyte activation is a common response to brain injury or disease, which is characterized by changes in morphology and functions, and astrocytes may shift in a stimulus-specific manner between the neurotoxic A1 phenotype and the neuroprotective A2 phenotype [30]. Evidence that has emerged in recent years has implicated astrocyte dysfunction in several pathological processes in PD. Alpha-syn released from damaged neurons is internalized by astrocytes and triggers an inflammatory response, impairment of the protein degradative system, mitochondrial dysfunction and endoplasmic reticulum (ER) stress. Mutations in several PD-associated genes expressed in astrocytes can also trigger or intensify astrocytic dysfunctions in addition to their roles in neurons. Whether and how astrocytic DJ-1 protein (encoded by *PARK7*) could be anti-inflammatory, glucocerebrosidase (GCase, encoded by *GBA1*), leucine-rich repeat kinase 2 (LRRK2, encoded by *LRRK2/PARK8*) and ATPase cation transporting 13A2 (ATP13A2, encoded by *ATP13A2*/*PARK9*) affect the protein degradation pathway, PTEN-induced putative kinase 1 (PINK1, encoded by *PINK1/PARK6*) and Parkin (encoded by *PRKN/PARK2*) are involved in mitochondrial dysfunction, or LRRK2, Parkin and calcium-independent phospholipase A2 (iPLA2, encoded by *PLA2G6*/*PARK14*) regulate ER stress in astrocytes have attracted much attention. Here, we review recent advances on the contributions of astrocytic dysfunction to the PD pathogenesis, especially from the perspective of transmitted α-syn. We also discuss how astrocytic gene factors modulate these pathological processes. Since several functions of astrocytes are disrupted in PD, novel approaches to replenishing functional astrocytes are discussed as potential PD therapeutics.

## Inflammatory response

A sustained inflammatory response with hallmarks of microglial and astrocyte activation plays significant roles in neurodegenerative diseases, including PD [31–33]. Astrocytes are considered to be an instrumental amplifier of neuroinflammation since proinflammatory mediators, such as interleukin (IL)-1α, tumor necrosis factor (TNF)-α and complement component 1q (C1q), and fragmented mitochondria released from activated microglia, can promote astrocytic activation to the A1 proinflammatory state and lead to further release of high levels of proinflammatory cytokines [30, 34, 35]. Prevention of microglial-mediated conversion of astrocytes to the A1 phenotype is neuroprotective in models of PD [36]. Although microglia are the prominent cells in the brain for inflammasome activation, astrocytes also can express and activate inflammasomes [37, 38]. Nod-like receptor protein 3 (NLRP3) inflammasomes are activated to produce IL-1β in the midbrain of PD model mice that overexpress mutant human A53T α-syn, although largely relying on microglial endocytosis of α-syn [39]. Astrocytes also have crosstalk with other cells, such as oligodendrocytes, endothelial cells and peripheral immune cells, during neuroinflammation [40].

Astrocytes with α-syn aggregates derived from iPSCs of PD patients show a more reactive state and secrete more proinflammatory cytokines (IL-6 and C–C motif chemokine ligand [CCL]-5) upon inflammatory stimulation [12]. Alpha-syn aggregates transmitted into astrocytes through intercellular transmission can upregulate the transcription of proinflammatory cytokines such as IL-1α, IL-1β, IL-6 and TNF-α [13, 23, 41, 42]. When astrocytes are exposed to transmitted α-syn, both receptor-mediated endocytosis and interactions with receptors on the cell membrane would occur. As pattern recognition receptors, toll-like receptor (TLR)-2 is involved in α-syn uptake in astrocytes, while TLR-4 is not [41, 43–45]. Both TLR-2 and TLR-4 are considered to mediate the proinflammatory effects of transmitted α-syn. The TLR-2-dependent accumulation of α-syn in astrocytes triggers neurotoxic proinflammatory responses of astrocytes through the nuclear factor kappa-B (NF-κB) and P38 mitogen-activated protein kinase signaling cascades, since anti-TLR-2 (a functional inhibitory antibody of TLR-2) administration blocks these responses [44]. Exposure to picomolar doses of physiological concentrations of α-syn oligomers over several days sensitizes TLR-4 responsiveness in astrocytes as well as in microglia [46]. In TLR-4-knockout astrocytes, the expression of proinflammatory cytokines triggered by α-syn, inducible nitric oxide synthase and cyclooxygenase-2 (COX-2), is significantly reduced [41, 43]. TLR-4 is most highly expressed within the human substantia nigra (SN), the most affected brain region in PD, reinforcing the participation of TLR4-mediated inflammatory responses in PD pathogenesis [46]. This evidence strongly suggests that extracellular α-syn can activate proinflammatory TLR-4 pathways in astrocytes, which may contribute to the pathogenesis of PD. However, there is evidence that rodent astrocytes actually lack TLR-4 [30]. Therefore, whether TLR-4 in astrocytes mediates the α-syn-induced neuroinflammation needs to be explored further. NF-κB is involved in TLR4-mediated inflammation [41, 43]; it also mediates the anti-inflammatory effects of nuclear receptor related-1 protein (Nurr1). As an important transcription factor, Nurr1 suppresses inflammatory gene expression in astrocytes and microglia [47, 48]. Alpha-syn aggregation in astrocytes results in decreased Nurr1, eventually leading to inadequate anti-inflammatory molecules and an inflammatory response [23]. Likewise, the β-arrestin2-mediated dopamine D2 receptor (Drd2) signal transduction has been regarded as a potential anti-inflammatory pathway in astrocytes [38, 49, 50]. However, overexpression of α-syn disrupts the anti-inflammatory role of Drd2 via β-arrestin2 in astrocytes [51].

Although α-syn could undoubtedly induce a proinflammatory phenotype in astrocytes, it causes production of relatively lower levels of proinflammatory cytokines than does TNF-α treatment [52]. Adaptably, astrocytes with accumulated α-syn have been revealed with a novel role as antigen-presenting cells in chronic neuroinflammation in PD. Postmortem brain tissues from PD patients show pathological, phosphorylated α-syn-loaded astrocytes that express high levels of major histocompatibility complex (MHC)-II. These astrocytes are situated close to both perivascular and infiltrated CD4^+^ T cells, indicating a cross-talk between astrocytes and T cells in PD brains [53]. In cultured astrocytes, α-syn exposure triggers surface expression of costimulatory molecules that are critical for T-cell activation, suggesting that astrocytes that have accumulated α-syn can more easily activate CD4^+^ T cells through the MHC-II pathway. The α-syn/MHC-II deposits could also be transferred between adjacent astrocytes via TNTs, indicating spreading of toxic protein aggregates and inflammation [53]. Consistent with this hypothesis, high-molecular-weight α-syn fibrils have been found to facilitate the acquisition of a reactive antigen (cross)-presenting phenotype by human iPSC-derived astrocytes, with upregulation of MHC gene expression and increased numbers of human leukocyte antigen (HLA) molecules at the cell surface. Small peptides derived from α-syn fibril degradation in astrocytes, rather than α-syn monomers, interact with MHC-II proteins and induce relocation of MHC molecules to the cell surface [52]. Interestingly, co-treatment of astrocytes with TNF-α and α-syn fibrils significantly decreased the effect of exposure to TNF-α or to α-syn fibrils alone, suggesting that TNF-α and α-syn fibrils compete to drive the immune reactive response of astrocytes to a proinflammatory phenotype or an antigen-presenting phenotype [52].

Gene factors have been suggested to modulate inflammation in astrocytes. Autosomal recessive mutations in *PARK7* encoding DJ-1 are linked to a rare, familial type of early-onset Parkinsonism. The DJ-1 protein can be highly upregulated in reactive astrocytes in the human PD brain [54, 55]. When stimulated by lipopolysaccharide (LPS), DJ-1-deficient astrocytes produce higher levels of proinflammatory mediators such as COX-2 and IL-6 [56], suggesting that DJ-1 might be a regulator of proinflammatory responses in astrocytes. Consistently, DJ-1 also inhibits the expression of proinflammatory mediators by interferon-gamma-treated astrocytes. This is mediated by the negative regulation of signal transducer and activator of transcription 1 (STAT1) activation, through enhancement of the interaction between STAT1 and its phosphatase SHP1 (Src-homology 2-domain-containing protein tyrosine phosphatase-1), and/or through enhancement of the expression of SOCS1 (Src-homology 2-domain containing protein tyrosine phosphatase-1), a negative regulator of STAT1, by miRNA-155 [57, 58]. Moreover, DJ-1-deficient astrocytes show decreased production and secretion of prostaglandin D2, an anti-inflammatory molecule, followed by less induced expression of anti-inflammatory heme oxygenase-1 in microglia [59]. Therefore, loss of DJ-1 function may contribute to neuroinflammation by aggravating the proinflammatory response and repressing the anti-inflammatory response of astrocytes. DJ-1 deficiency also causes defects in neurotrophic factor production and reduces CCL2 expression in astrocytes under conditions of brain damage, attenuating monocyte infiltration into the damaged brain and resulting in delayed repair of brain injury, a situation that might contribute to the development of PD [60, 61]. Based on these findings, astrocytic DJ-1 overexpression is proposed to be neuroprotective. Recently, a transgenic zebrafish line with increased astrocytic DJ-1 expression was generated; these zebrafish were rescued from the loss of dopaminergic neurons and motor deficits under exposure to 1-methyl-4-phenylpyridinium. A large group of proteins associated with inflammation, redox regulation and mitochondrial respiration were observed to be upregulated in DJ-1-overexpressing astrocytes [62]. DJ-1 overexpression in astrocytes is also neuroprotective in rotenone-induced PD rats, as indicated by a marked reduction of PD-like pathology including decreased loss of dopaminergic neurons and decreased neuroinflammation. Activated microglia and astrocytes in the rat SN are both reduced, indicating that DJ-1 in astrocytes is sufficient to protect neighboring neurons from inflammatory damage, although DJ-1 plays a neuroprotective role in multiple ways [63].

## Protein degradation dysfunction

Protein degradation dysfunction is believed to be a significant factor that contributes to the accumulation of damaged and abnormally modified proteins in neurodegenerative diseases, including PD. In astrocytes, as in other cells, there are two crucial pathways that mediate the refolding or removal of abnormal proteins: the ubiquitin–proteasome system (UPS) and the autophagy–lysosomal pathway (ALP) [64, 65]. Dysfunction of the protein degradation pathways in astrocytes might lead to the accumulation of protease-resistant misfolded and aggregated proteins.

As mentioned above, α-syn can be transferred from neurons to astrocytes and from astrocytes to astrocytes. Interestingly, less α-syn is transferred from astrocytes to neurons than from neurons to astrocytes. In contrast to neurons, which may contain fibrillar α-syn that resists degradation for several days, astrocytes are able to efficiently degrade α-syn, indicating that astrocytes play a major role in the degradation rather than in the spreading of α-syn aggregates [66]. Once inside astrocytes, α-syn oligomers or fibrils colocalize with lysosomal-associated membrane protein (LAMP) 1, suggesting a following delivery to lysosomal compartments for degradation [18, 66]. Consistent with this, a recent study of iPSC-derived dopaminergic neurons and astrocytes showed that the astrocytes rapidly internalize α-syn and exhibit higher lysosomal degradation rates than neurons [67]. It has been demonstrated that bafilomycin A1, a lysosomal inhibitor, increases the formation of detergent-insoluble α-syn in astrocytes [13]. The proteasome inhibitor MG132 and the autophagy inhibitor 3-methyladenine have similar effects; conversely, recovery of the degradative ability of astrocytes mediated by ginkgolide B and bilobalide, two functional components isolated from *Ginkgo biloba*, reduces the levels of intracellular α-syn engulfed by astrocytes [68], suggesting that both the UPS and the autophagy–lysosomal pathway are involved in α-syn degradation within astrocytes. Moreover, coculture of neurons and astrocytes results in decreased accumulation of α-syn in neurons and consequently diminished interneuronal transfer of α-syn [67]. This observation suggests that astrocytes may confer neuroprotection on dopaminergic neurons by relieving intraneuronal accumulation of α-syn and clearing excessive extracellular toxic α-syn. Alpha-syn released from neurons could also activate microglia, which in turn engulf α-syn into autophagosomes for degradation via the TLR4-NF-κB-p62-mediated selective autophagy (termed synucleinphagy) [69]. In mice treated with human α-syn preformed fibrils (PFFs), exosomes released by microglia can facilitate the transfer of exogenous human α-syn to neurons, resulting in protein aggregation in the recipient neurons [70, 71]. Considering that astrocytes and microglia both play crucial roles in the intercellular transmission of α-syn, it is speculated that there may be associations between astrocytes and microglia in synucleinopathy. This hypothesis has recently been verified in a study which found that the crosstalk between astrocytes and microglia reduces the intracellular deposits of α-syn in these cells under exposure to α-syn PFFs. Astrocytes are less effective than microglia in degrading the ingested α-syn but transfer a high proportion of their intracellular α-syn to microglia, while microglia participate in degradation and hardly transfer α-syn to astrocytes. The transfer of α-syn from astrocytes and microglia can be achieved by secretory mechanisms and through direct membrane contact via TNTs and other membrane structures [72].

Upon extensive uptake of α-syn aggregates beyond the degradative capacity, astrocytes may develop intracellular deposits. In vitro data showed that after 24-h exposure to α-syn oligomers followed by culture in α-syn-free medium, a majority of the α-syn inclusions remained in the astrocytes even 12 days afterward, indicating that their degradation was not complete [18, 20]. In vivo studies have also shown that intracerebral injection of both soluble and aggregated α-syn induces extensive α-syn inclusion in astrocytes [73, 74]. Recently, a small heat shock protein, αB-crystallin, has been found to be a natural inhibitor of astrocyte autophagy. By selectively inhibiting the process of autophagy, overexpression of αB-crystallin in astrocytes leads to the accumulation of α-syn aggregates in the brains of transgenic mice expressing the human α-syn A30P mutant [75]**.** Interestingly, αB-crystallin is markedly upregulated in the SN of PD patients, where it is present in glial cell inclusions. In a neurotoxin-induced mouse model of PD, reactive astrocytes have also been found with increased expression of αB-crystallin [76]**.** These results suggest that aberrant increases of αB-crystallin induced in the initial phase of PD inhibit astrocytic autophagy, leading to more severe α-syn aggregation or even the formation of cell inclusions. Consistent with these hypotheses, postmortem brain analysis showed that α-syn-immunoreactive inclusions appear frequently in astrocytes in various brain regions of patients with PD, including the SN, the cerebral cortex, the olfactory bulb and other regions [77–79]. Clinical data also indicate that the development of α-syn-immunoreactive astrocytes in the forebrain parallels the stages of intraneuronal pathology in sporadic PD, raising the possibility that α-syn aggregation in astrocytes might be related to PD progression [78]. In addition, astrocytes derived from human ESCs fail to degrade excessive α-syn oligomers, and α-syn is deposited in the trans-Golgi network, inducing TNT formation. The TNTs mediate transfer of intracellular α-syn to healthy astrocytes, which may be a rescue mechanism for damaged astrocytes. However, TNTs may also accelerate the transfer of toxic α-syn between astrocytes [20]. Overall, astrocytes first attempt to degrade engulfed α-syn but ultimately fail to degrade excessive α-syn; they then transfer some α-syn to neighboring astrocytes, extending the overall development of synucleinopathy. The transfer of α-syn from astrocytes to neurons, though rarely occurring, may be detrimental to neurons, since under treatment with Lewy body extracts from PD brains, the uptake and transport of α-syn aggregates from astrocytes to neurons is sufficient to lead to neuronal death [16].

A number of genetic factors have been suggested to affect the protein degradation pathway in astrocytes, causing an impairment of protein clearance. Mutations in astrocytic *LRRK2 (PARK8)*, *GBA1* and *ATP13A2 (PARK9)* can reduce the astrocytic degradation capability to various extents and aggravate α-syn accumulation, which may promote PD development and progression.

*PARK8 (LRRK2)* encodes a large protein LRRK2 with dual kinase and GTPase activity. *LRRK2* missense mutations causing abnormal LRRK2 kinase activity are the most common cause for autosomal dominant PD [80–82]. Activation of the LRRK2 kinase activity, even independent of mutations, also contributes to the pathogenesis of idiopathic PD [83]. LRRK2 is expressed in astrocytes in the human brain [84] and localized primarily in lysosomes to regulate lysosome size, number and function. However, G2019S, R1441C and Y1699C mutations in *LRRK2* in astrocytes result in enlarged lysosomes, inhibit lysosomal degradation of long-lived proteins and reduce lysosomal pH by increasing the LRRK2 kinase activity [85]. In comparison to normal astrocytes that usually display low levels of α-syn, the Parkinson's iPSC-derived astrocytes carrying the *LRRK2* G2019S mutation show progressive endogenous α-syn accumulation, dysfunctional chaperone-mediated autophagy and impaired macroautophagy [86]. When cocultured with healthy dopaminergic neurons, astrocytes with *LRRK2* G2019S mutation transfer α-syn to neighboring neurons, resulting in α-syn accumulation and neurodegeneration; this suggests that the *LRRK2* mutation in astrocytes can compromise neuronal survival during PD pathogenesis [86]. The *LRRK2* G2019S mutation can also reduce the ability of astrocytes to clear extracellular α-syn aggregates. RNA sequencing data demonstrate that matrix metallopeptidase 2, which has been shown to degrade extracellular α-syn aggregates [87], is downregulated in Parkinson's iPSC-derived astrocytes with the *LRRK2* G2019S mutation [88]. Moreover, astrocytes harboring the *LRRK2* G2019S mutation display deficits in an actin-binding protein annexin A2, which is also a novel player in α-syn internalization by astrocytes. As a consequence, the overall capacity of the endolysosomal pathway to clear extracellular fibrillar α-syn is impaired, although the *LRRK2* mutation has no influence on lysosomal degradation of extracellular fibrillar α-syn [89].

The *GBA1* gene encodes the lysosomal enzyme GCase, which catalyzes the conversion of glucosylceramide to glucose and ceramide. *GBA1* mutations can cause GCase deficiency and a lysosomal storage disorder Gaucher disease (GD). Patients with GD and heterozygous carriers of *GBA1* mutation are both at an increased risk of developing PD [90, 91]. It has been reported that mutations in *GBA1* can structurally change the GCase protein in neurons, resulting in decreased enzyme activity and lysosomal accumulation of substrates, which, in turn, alter lysosomal degradation and lead to increased α-syn aggregation [92–94]. *GBA1*-deficient mice show enhanced α-syn expression and reactive astrocytes in the nigrostriatal pathway, suggesting that the astrocytes play important roles in the pathogenesis of PD [95]. In a mouse model with a *GBA1* mutation, the autophagic machinery and the proteasomal machinery are defective in both neurons and astrocytes [96]. In a recent study, astrocytes and dopaminergic neurons were generated from iPSCs derived from fibroblasts from patients with GD. When the astrocytes were cocultured with the dopaminergic neurons obtained from patients with this *GBA1* mutation, the excessive α-syn released by neurons was endocytosed by astrocytes, where it was colocalized with the lysosomal marker LAMP2, suggesting that astrocytes attempted to degrade α-syn, especially the higher-molecular-weight species of α-syn. When these astrocytes were treated with monomeric or fibrillar α-syn, they showed increased levels of aggregated α-syn. These data indicate that astrocytes carrying the *GBA1* mutation might experience both more transmission of excessive α-syn from *GBA1* mutant dopaminergic neurons and accumulation of more aggregates of α-syn due to inadequate degradation [42]. However, there is also evidence suggesting that the aforementioned *GBA1* mutation in astrocytes has no effect on α-syn degradation. A recent study has indicated that astrocytes degrade α-syn PFFs over time, as expected; however, the rate of degradation of α-syn PFFs in these cells is not decreased after treatment with a GCase inhibitor, suggesting that the ability of the astrocytes to degrade exogenous α-syn is not impaired by GCase inhibition [97]. Consistent with this, the *GBA1* D409V mutation also has no effect on α-syn degradation by astrocytes. The D409V mutation has been reported to cause lysosome impairment in primary mouse astrocytes, as indicated by decreased number of lysosomes, increased luminal pH of lysosomes and a loss of lysosome protease activity. However, the mutant astrocytes degrade monomeric α-syn or PFFs engulfed by them at the same rate as WT astrocytes do [98]. Based on the above investigations, it remains still controversial whether the *GBA1* mutations in astrocytes are crucial to α-syn accumulation. While GCase deficiency is not sufficient to induce α-syn accumulation and neurodegeneration in vivo [97, 99], inhibition of GCase in the presence of α-syn insults can cause combinatorial α-syn pathology [97]. Therefore, the combined actions of *GBA1* mutations in astrocytes and neurons need to be elucidated further.

*PARK9* encodes the transmembrane lysosomal P5-type ATPase ATP13A2, and missense or truncation mutations of *PARK9* impair lysosomal function, resulting in autosomal recessive levodopa-responsive early-onset Parkinsonism, also known as Kufor-Rakeb syndrome [100, 101]. Disturbance of ATP13A2 function in neurons is believed to induce lysosomal impairment, α-syn accumulation and mitochondrial dysfunction [102, 103]. Similarly, knockout of ATP13A2 in astrocytes increases lysosomal membrane permeabilization and induces the outflow of cathepsin B, a lysosomal proteolytic enzyme, thereby severely impairing the lysosomal function. Moreover, the secretion of cathepsin B further activates the NLRP3 inflammasome, suggesting that ATP13A2 mediates the communication between lysosomes and neuroinflammation in astrocytes [104]. More importantly, ATP13A2 deficiency decreases the rates of α-syn internalization and degradation in astrocytes, resulting in increased accumulation of α-syn in neurons and interneuronal α-syn transmission. Therefore, a lack of ATP13A2 in astrocytes can result in failure to protect dopaminergic neurons from α-syn accumulation and propagation, thereby potentially contributing to PD pathology [67].

Finally, functional crosstalk among *LRRK2*, *ATP13A2* and *GBA1* in astrocytes has been suggested. Increased expression of ATP13A2 has been observed in primary *LRRK2* G2019S-mutant astrocytes; this might act as a compensatory mechanism to normalize deficits in lysosomes induced by the *LRRK2* mutant [85]. Similarly, *GBA1*-mutant astrocytes exhibit downregulation of endogenous LRRK2 kinase activity, an effect that might represent a compensatory effort of astrocytes to attenuate the effects of *GBA1* mutations on lysosomal function. However, the compensatory effects are not sufficient to fully rescue the defects in lysosome function; LRRK2 kinase inhibition in *GBA1* D409V knock-in mouse astrocytes is still beneficial, and normalizes the *GBA1* mutant-induced phenotypes [98]. Overall, this evidence highlights the importance of lysosomal function in astrocytes in PD pathogenesis.

## Mitochondrial dysfunction

Astrocytes are responsible for 20% of the total oxygen consumption of the brain, although they rely heavily on glycolysis for energy production [105]. Essential energy output occurs during oxidative phosphorylation within astrocytic mitochondria, leading to the production of ATP; thus, proper mitochondrial function is important for maintaining normal physiological functions of astrocytes. In neurodegenerative diseases and injuries, astrocytic mitochondrial dysfunction and insufficient energy production commonly occur with astrocyte activation, leading to calcium dysregulation, overproduction of reactive oxygen species, and cell death cascades in astrocytes [106].

Emerging data have indicated that pathogenic α-syn, a significant factor that induces astrocyte activation, is also involved in astrocytic mitochondrial dysfunction. When oligomers formed from recombinant human α-syn are internalized by human primary astrocytes, they are localized to mitochondria and cause reduced oxygen consumption [17]. Similarly, exposure of cultured astrocytes to α-syn oligomers for 24 h induces changes in mitochondrial morphology (causing the mitochondria to exhibit a fragmented pattern), disturbance of mitochondrial fission–fusion dynamics and a decrease in ATP level [18]. Accumulation of α-syn aggregates in the trans-Golgi network region of human ESC-derived astrocytes can also impair mitochondrial dynamics and lead to widespread mitochondrial fragmentation [20]. This could be pattern-specific as oligomeric, but not monomeric or fibrillar forms of α-syn, induce mitochondrial dysfunction in astrocytes and significantly increase extracellular hydrogen peroxide production by the cells [14]. Correspondingly, the phenotypes of mitochondria in α-syn-ingesting astrocytes can be restored by addition of oligomer-selective antibodies that reduce the accumulation of α-syn in cultured astrocytes [107]. More recently, high-molecular-weight α-syn fibrils have also been reported to decrease the ATP-generating mitochondrial respiration and induce mitochondrial dysfunction in human iPSC-derived astrocytes [52]. Ultrastructural and functional mitochondrial impairment has also been observed in mesencephalic astrocytes from transgenic mice overexpressing doubly mutated human α-syn [108, 109]. When these astrocytes were cocultured with neurons, they failed to exert neurotrophic effects on neuronal differentiation, suggesting that the mitochondrial dysfunction induced by mutant α-syn overexpression in astrocytes may prevent their supportive role for neurons [108].

Astrocytic mitochondria may be a significant source of energy for neurons. Cultured astrocytes can shed large membrane-bounded vesicles that contain mitochondria, lipid droplets and ATP into the extracellular space [110]. The release of extracellular particles containing functional mitochondria can rescue neurons that have been subjected to oxygen–glucose deprivation by allowing recovery of energy production and restoration of their viability. This has been observed in a mouse model of focal cerebral ischemia; in those experiments, mitochondria released by astrocytes entered adjacent neurons and supported their viability and recovery after stroke [111]. Recently, a similar neuroprotective mechanism of astrocytic mitochondria in PD has been elucidated. Dopaminergic neurons that survive after 6-hydroxydopamine (6-OHDA) treatment have higher energy requirements to compensate for the lost neurons. However, the surviving neurons are no longer able to enhance their function or to compensate when the mitochondria in the surrounding astrocytes are defective, suggesting that astrocytic mitochondria provide important energy support for repair of early deficits in the SN [112]. In a coculture system of dopaminergic neurons and astrocytes (both generated from human iPSCs), the astrocytes spontaneously release functional mitochondria into the medium; the transferred functional mitochondria are internalized by the injured neurons via a phospho-p38-dependent pathway, thus restoring the rotenone-induced ATP depletion and preventing neurodegeneration. The neuroprotective effects are abolished when the medium is depleted of mitochondria by ultrafiltration [113]. These data indicate that astrocytes may supply energy to neurons in the form of ATP or may directly transfer functional mitochondria to neurons to limit the progressive loss of dopaminergic neurons. Therefore, when astrocytes experience mitochondrial dysfunction induced by α-syn, their potential to support dopaminergic neurons may diminish. However, healthy astrocytes can transfer mitochondria to stressed α-syn-containing astrocytes, by releasing them into the extracellular space and transmission through TNTs, indicating an astrocyte-to-astrocyte rescue mechanism [20]. Recently, astrocytes activated by fragmented mitochondria released from microglia have been shown not only to exhibit pathological mitochondrial fragmentation and mitochondrial dysfunction, but also to release dysfunctional mitochondria that act as mediators of neurotoxic signaling, inhibiting neuronal mitochondrial respiration and substantially increasing neuronal cell death [34]. Whether the dysfunctional mitochondria induced by α-syn in astrocytes are also released as neurotoxic signals needs to be studied in future.

In astrocytes exposed to α-syn oligomers, the mitophagy pathway is activated in order to degrade the dysfunctional mitochondria in autophagosomes [20]. Eukaryotic cells are capable of degrading their own dysfunctional mitochondria via autophagy in a process called mitophagy. Mitophagy is a critical component of mitochondrial quality control and is necessary for the elimination of dysfunctional mitochondria to maintain cellular respiration. Mitophagy deficiency has been recognized as a key contributor to PD, largely from the perspective of neurons [114]. Interestingly, after α-syn exposure for 6 days, despite the remaining fragmented mitochondria, the number of autophagosomes is reduced to normal levels, indicating the initiation but eventual halting of the mitophagy pathway [20]. This suggests that ineffective mitophagy might be related to the increased number of fragmented mitochondria observed after exposure of astrocytes to α-syn. Recently, knockout of astrocytic Kir6.1 has been shown to inhibit mitophagy and to cause an increase in the accumulation of damaged mitochondria [115]. Kir6.1, which is prominently expressed in astrocytes, is a pore-forming subunit of ATP-sensitive potassium channels, which are unique channels that couple cell metabolism to cell membrane potential [116, 117]. More importantly, knockout of the astrocytic Kir6.1 leads to increased degeneration of dopaminergic neurons in the SNpc and more severe motor dysfunction in MPTP-induced PD models. Restoration of astrocytic mitophagy rescues the deleterious effects of astrocytic Kir6.1 ablation on mitochondrial function and dopaminergic neuron survival [115]. This suggests that astrocytic mitophagy is important for neuronal survival. Astrocytes have also been found to be the host for dopaminergic neuronal mitophagy, that is, spheroid-mediated transmitophagy. Damaged mitochondria in degenerating dopaminergic neurons induced by 6-OHDA are retained in spheroids, where the mitophagy process is initiated but not completed. The spheroids were then penetrated by astrocytic processes, and the damaged mitochondria were further degraded in astrocytes. The neuron-astrocyte transmitophagy could be critical for preventing the release of damaged mitochondria to the extracellular medium and the subsequent detrimental activities [118, 119].

Mutations in *PARK6*, which encodes PINK1, and in *PRKN/PARK2*, which encodes Parkin, both contribute to autosomal recessive PD. As a protein kinase, PINK1 phosphorylates a conserved serine at amino acid 65 (pS65) in ubiquitin and Parkin. As an E3 ubiquitin ligase, Parkin cooperates with E1 ubiquitin-activating enzymes and E2 ubiquitin-conjugating enzymes of the UPS to degrade targeted proteins. PINK1 can selectively stabilize impaired mitochondria, thereby activating Parkin, which then targets the dysfunctional mitochondria for degradation by autophagy (mitophagy) to maintain cellular respiration [120, 121]. Activation of PINK1 by valinomycin, a chemical agent that causes mitochondrial injury, occurs predominantly in astrocytes rather than in neurons, microglia or oligodendrocytes; this suggests that astrocytes represent a key experimental system in which to address the question of why PINK1 deficiency invariably leads to PD-related neurodegeneration [81]. Parkin deficiency in astrocytes is sufficient to cause presence of a higher number of structurally altered mitochondria in the cells [108]. More importantly, the decrease in ATP-generating mitochondrial respiration induced by high-molecular-weight α-syn fibrils is exacerbated in Parkin-variant-containing PD astrocytes [52]. Reduced astrocyte proliferation (lower levels of glial fibrillary acidic protein (GFAP)) has been found in human brains and in iPSC-derived midbrain organoids harboring Parkin mutations [122]. Like Parkin deficiency, PINK1 deficiency can also attenuate astrocyte proliferation and inhibit GFAP-positive astrogliogenesis [123, 124]. The appearance of dysfunctional mitochondria in Parkin- and PINK1-deficient astrocytes might be related to mitophagy defects, and this might further lead to a decrease in astrocyte proliferation, causing sparser distribution of GFAP-positive astrocytes in the SN and possibly contributing to the development of PD due to the delay of astrocyte-mediated repair of the brain microenvironment [122–124].

## ER stress

The ER plays a central role in sensing cellular stress based on protein unfolding/misfolding in its lumen (known as ER stress), with consequent activation of the unfolded protein response (UPR). Astrocytes share the same conserved UPR proteins and pathways, and an increasing level of unfolded proteins inside the ER lumen provokes the dissociation of grp78/BiP (78-kDa glucose-regulated protein) from three ER transmembrane receptors—PKR-like endoplasmic reticulum kinase (PERK), activating transcription factor 6 and inositol-requiring enzyme 1—thereby initiating the UPR [125]. Although the UPR is activated in an attempt by the cell to restore ER functionality by increasing its protein-folding capacity and inducing a transient reduction in the flux of proteins entering the ER through these pathways [126], sustained UPR activation resulting from prolonged ER stress in astrocytes might promote further pathological processes contributing to PD.

It is well accepted that ER stress is an important mediator of α-syn toxicity in neurons since it is highly likely to cause accumulation of unfolded/misfolded α-syn in the ER [127–129]. Intercellularly transmitted α-syn may be an inducer of ER stress in neurons [130, 131]. ER stress could also be triggered in astrocytes by α-syn overexpression or excessive uptake. In highly purified rat primary astrocyte lines overexpressing wild-type or mutant α-syn (A30P and A53T), both wild-type and mutant α-syn trigger ER stress in the astrocytes, with the presence of Golgi apparatus fragmentation, and the subsequent activation of a CHOP (CCAAT-enhancer-binding protein homologous protein)-mediated pathway eventually leads to apoptosis [132]. As mentioned above, the ESC-derived astrocytes cannot degrade excessive α-syn oligomers, resulting in deposition of α-syn in the trans-Golgi network and increased pressure in the ER lumen [20]. In this case, ER stress is very easily triggered. Treatment of primary astrocytes with monomeric or oligomeric α-syn also induces increased mRNA and protein levels of ER stress markers [133].

The fact that the inflammatory response can be triggered by transmitted α-syn in astrocytes has been discussed previously, and ER stress is believed to directly elicit inflammation. The PERK signaling pathway is believed to closely link astrocytic ER stress to inflammation. The activation of the Janus kinase 1 (JAK1)/PERK/STAT3 axis during ER stress can induce the expression of IL-6 and several chemokines [134, 135]. RNA sequencing in primary murine astrocytes has shown that approximately 10% of ER stress-induced gene expression is regulated by JAK1, and JAK1 is also required for the expression of a subset of PERK-dependent genes. PERK haploinsufficiency or partial PERK inhibition reduces the ER stress-induced inflammation (as indicated by decreased IL-6, CCL2 and CCL20 expression), and complete loss of PERK blocks the expression of canonical PERK-dependent UPR genes [136]. The inflammatory reaction induced by ER stress in astrocytes can subsequently stimulate microglia to acquire an inflammatory M1-like phenotype via paracrine signaling; IL-6 and oncostatin M produced by activated microglia could further enhance the inflammatory response of ER-stressed astrocytes, and these effects can be attenuated through inhibition of PERK [134, 136]. The interaction of ER-stressed astrocytes with activated microglia may contribute to the excessive and non-resolving neuroinflammation associated with PD. ER-stressed astrocytes also fail to support synaptogenesis. Chronic PERK branch activation drives a distinct ‘‘UPR’’-reactivity state in primary cultured astrocytes, leading to an altered secretome with reduced neurotrophic factors and constituents of the extracellular matrix, all of which are important for synaptic formation and maintenance [137]. The ER stress triggered in astrocytes receiving transmitted α-syn thus not only directly elicits changes in astrocytic function but also contributes to neurodegeneration by influencing adjacent microglia and neurons.

Although astrocytic ER stress is detrimental, mild ER stress might be beneficial if it decreases the responsiveness of astrocytes to inflammatory stimuli. Relative to this point, benign and mild ER stress in primary cultured astrocytes induced by low concentrations of tunicamycin (a pharmacological ER stressor) can profoundly attenuate the subsequent LPS-induced astrocytic inflammatory responses (IL-1β and IL-6 production). Preexposure to nonlethal doses of tunicamycin alleviates the LPS-induced overactivation of astrocytes, blood–brain barrier (BBB) impairment and cognitive ability dysfunction in vivo, while the ER stress inhibitor sodium 4-phenylbutyrate reverses the protective effects both in vitro and in vivo [138]. Astrocytes with mild exposure to ER stress are also beneficial to the surrounding cells. When neurons are treated with the astrocyte-conditioned medium, they display resistance to acute ER stress induced by thapsigargin (a pharmacological ER stressor), including lower levels of proapoptotic factors and significantly lower caspase-3 activity [139]. Compared to the opinion that ER stress in astrocytes is a response that primarily contributes to neurodegeneration and should therefore be broadly attenuated, it is more scientific to hold the view that the role of ER stress in astrocytes is complicate and can be influenced by multiple factors such as the severity, the duration and the concentration of the insult.

A recent study has reported for the first time that ER stress might be transmissible and that UPR markers in resting astrocytes and neuronal cells are upregulated when the cells are cultured in conditioned medium from astrocytes that have been subjected to pharmacological ER stressors. However, the functional molecules mediating this process are still unknown; the active substance is assumed to be lipid in nature since it is resistant to proteinase K and has lipid-like but not microparticle-like properties [139]. However, there are opinions that the pharmaca-based cell culture protocols are unsuitable for use in studies of UPR transmission because carryover of the pharmaca is a strong confounding factor [140, 141]. This is supported by the fact that no UPR transmission is observed in vitro when nonpharmacological UPR induction paradigms involving nutrient deprivation or genetic tools are used. This not only suggests that methodological artifacts might occur during pharmacological induction of the UPR, but also raises the question of whether UPR transmission is a real phenomenon [140]. Therefore, whether astrocytes can transmit ER stress to neighboring cells remains to be explored.

Astrocytic ER stress might be a coordinator in the pathogenesis of PD patients with *LRRK2* and *PRKN* mutations. ER stress markers are increased to higher levels in astrocytes from an *LRRK2*-G2019S mutant (LRRK2-GS-Tg) compared to those in wild-type cells after α-syn treatment. Further experiments showed that *LRRK2*-G2019S suppresses sarco/endoplasmic reticulum Ca^2+^-ATPase activity, leading to the Ca^2+^-mediated ER stress and increasing the ER stress-mediated astrocyte death [133]. This suggests that on the basis of α-syn-induced ER stress, *LRRK2* mutations might further aggravate the consequences of ER stress in astrocytes. A transgenic in vivo model expressing the *LRRK2*-G2019S mutant exhibits high levels of dopaminergic neuronal loss [142, 143]. There are no differences in ER stress response between wild-type and *LRRK2*-G2019S neurons. However, when these neurons are cocultured with *LRRK2*-G2019S astrocytes, α-syn treatment causes more neuronal damage and death. These data suggest that the functions of *LRRK2*-G2019S in neurons and astrocytes differ with respect to the ER stress; specifically, neurodegeneration in *LRRK2*-G2019S mice might be secondary to abnormal astrocytes rather than a direct effect of *LRRK2*-G2019S on neurons [133]. This reinforces the importance of *LRRK2* G2019S-induced ER stress in astrocytes to the development of PD; however, considering that the *LRRK2* mutation also definitely impairs protein degradation pathways in astrocytes, it is difficult to determine which pathway is a dominant one. The E3 ubiquitin ligase Parkin, encoded by *PRKN,* is believed to mediate the ER stress signaling, especially in astrocytes. Although constitutive Parkin expression is lower in astrocytes than in neurons, a selective increase in astrocytic Parkin expression may occur during the UPR [144]. As a Parkin substrate, the nucleotide-oligomerization domain receptor 2 (NOD2) can be degraded by Parkin in a proteasome-dependent manner, and Parkin deficiency is also associated with the stress-induced elevation of NOD2. As a cytosolic receptor, NOD2 can integrate ER stress and inflammation [145]. In response to ER stressors, astrocytes with *PRKN* knockdown display exaggerated ER stress, c-Jun N-terminal kinase activation and cytokine release, as well as reduced neurotropic factor expression. NOD2 depletion rescues these responses in *PRKN* KO astrocytes, suggesting that Parkin degrades NOD2 and thereby blunts the astrocytic ER stress [146]. More importantly, *PRKN* KO astrocytes augment the injury of dopaminergic SHSY5Y cells and primary neurons induced by ER stressors. In contrast to ER stressors, traditional neurotoxic stressors (6-OHDA and MPTP) appear not to be linked to Parkin dysfunction, as the level of neuronal cell death is unchanged when wild-type or *PRKN* KO astrocytes are exposed to these stressors. These data indicate that the specific role of Parkin in astrocytes is primarily to suppress ER stress and thereby to maintain neuronal integrity [146].

*PARK14* (*PLA2G6*) encodes iPLA2, an enzyme that hydrolyzes phospholipids, generating free fatty acids and lysophospholipids. Homozygous mutation of *PLA2G6* is linked to early-onset PD [147, 148]. Mice with mutation display common PD pathology and early-onset parkinsonism phenotypes [149]. A series of PD-related events including ER stress, disturbed calcium homeostasis and mitochondrial dysfunction, are seen in these mice as well as iPSC-derived dopaminergic neurons with *PLA2G6* mutations [149, 150]. A more recent study shows that the loss of iPLA2 in *Drosophila* disrupts the membrane lipid equilibrium due to acyl-chain shortening in phospholipids, leading to chronic ER stress, yet unaffected mitochondrial activity. Meanwhile, correction of phospholipid composition alleviates the ER stress and the neuronal phenotypes [151]. Considering ER is the compartment orchestrating protein synthesis and folding, as well as synthesis of lipids and maintenance of Ca^2+^ homeostasis, we suppose that ER stress could be a major pathogenic mechanism underlying the *PLA2G6*-associated neurodegeneration. As extracellular α-syn can be endocytosed and induce ER stress [131], whether abnormal membrane lipid equilibrium induced by *PLA2G6* mutations in astrocytes in the presence of transmitted α-syn could aggravate ER stress and neurodegeneration needs to be further explored.

## Iron metabolism disturbance

Astrocytes express almost all iron transporters and various types of proteins involved in iron metabolism [152–154], and play a significant role in iron uptake, storage and release, thereby maintaining balanced iron metabolism in the brain. Astrocytic and microglial dystrophy/senescence that occurs with aging appears to disrupt metal homeostasis, including iron homeostasis, and this renders the brain susceptible to neurodegenerative diseases [155]. How astrocytes contribute to iron metabolism disturbance in PD has been discussed recently [156].

In primary cultured astrocytes, extracellularly transmitted α-syn has no effect on the expression of astrocytic iron metabolism-related proteins. However, when these astrocytes are overloaded with iron, a markedly decreased hepcidin-to-ferritin ratio is observed. The low hepcidin-to-ferritin ratio efficiently reflects the situation in iron-overloaded astrocytes that encounter extracellular α-syn; in these cells, the iron-releasing phenotype is facilitated, and this might be detrimental to neighboring neurons when the astrocytes are exposed to both elevated iron levels and transmitted α-syn [157]. Although hepcidin in astrocytes does not respond to transmitted α-syn, hepcidin secreted by astrocytes can regulate iron intake at the BBB by controlling ferroportin (FPN) in brain microvascular endothelial cells [154, 158, 159]. A recent study revealed that overexpression of hepcidin in astrocytes significantly reduces iron levels in the cortex and hippocampus in an APP/PS1 mouse model of Alzheimer's disease, especially the iron content of neurons, and suppresses the iron accumulation-induced oxidative stress and neuroinflammation in the cortex and hippocampus. Further, the model mice are partially protected from neuronal loss and cognitive decline, and the formation of Aβ plaques in the cortex and hippocampus is partially alleviated [160]. In PD models induced by rotenone and 6-OHDA, overexpression of hepcidin in neurons and glia using a virus-based strategy prevents dopaminergic neurodegeneration in the SN, motor deficits, as well as cellular/mitochondrial iron accumulation and mitochondrial deficits in neurons [161]. Although this study does not specifically discuss astrocytic hepcidin function, we speculate that the elevated expression of hepcidin in astrocytes might restrict iron transport from peripheral organs to the brain, a situation that may be beneficial in alleviating brain iron accumulation and PD symptoms. Modulation of astrocytic iron metabolism has been reported to mediate the neuroprotective effects of estrogen, considering that there is usually a lower risk of PD in females [162]. In primary cultured astrocytes, estrogen increases the expression of FPN and divalent metal transporter 1 by inducing the expression of hypoxia-inducible factor-1 alpha. Enhancement of the iron transport ability of astrocytes leads to discharge of excessive iron to other tissues such as blood vessels, which may lead to a decrease of iron levels in the brain [163]. It is difficult to decide whether astrocytic iron transport should be suppressed to limit peripheral iron import or whether it should be enhanced to facilitate excessive iron export. However, the data discussed here suggest that the regulation of astrocyte iron metabolism and the existence of possible iron metabolism disturbances in PD are worthy of further investigation.

The homeostatic iron regulator (*HFE*) gene variant (H63D), as a disease-modifier in PD, has been extensively studied. This *HFE* variant causes impaired function of HFE in limiting the cellular iron uptake, thereby promoting the brain iron overload and consequently generating oxidative stress in the brain [164, 165]. Recently, increased iron storage within L-ferritin and activation of the nuclear factor E2-related factor 2 (Nrf2) antioxidant defense system have been found in H67D (equivalent of the human H63D variant)-*HFE* astrocytes after paraquat exposure. Compared with wild-type astrocytes, these astrocytes exhibit limited senescence and mediate neuroprotection against the paraquat-induced neurotoxicity. These findings suggest that the oxidative stress induced by altered iron homeostasis in *HFE*-variant astrocytes promotes the development of adaptive mechanisms that help the astrocytes protect themselves and the surrounding neurons from external stressors [166]. A recent study measured the expression of genes in cortical regions with iron deposition from PD patients and found that sets of genes whose expression correlates significantly with cortical iron deposition are predominantly expressed in astrocytes and glutamatergic neurons [167]. Therefore, the role of genetic modulations in astrocytic iron homeostasis may attract more attention in the future.

## Replenishment of functional astrocytes

As mentioned above, astrocytic dysfunction is detrimental to surrounding neurons and contributes to the pathogenesis of PD. Astrocytes are believed to protect nigrostriatal dopaminergic neurons, at least during early neurodegeneration [168]. Therefore, therapeutic approaches that employ functional astrocytes to replace dysfunctional astrocytes and in part convert them to dopaminergic neurons may be promising to slow down or even halt the progression of PD.

In an established neurotrophic host brain environment, neural progenitor cells (NPCs) cocultured with ventral midbrain-derived astrocytes can be induced to differentiate into dopaminergic neurons with enhanced neuronal maturity, higher levels of midbrain-specific markers, and greater resistance to a toxic stimulus compared with control cultures. These neuroprotective effects are further increased when the astrocytes coexpress Nurr1 and Forkhead Box A2. The cografting of astrocytes also potentiates the therapeutic effects of NPC transplantation in a hemiparkinsonian rat model for at least 6 months after transplantation [169]. More recently, grafting of ventral midbrain-derived astrocytes into the SN has been explored in aged MPTP mice after the onset of motor symptoms. The mice exhibit a time-dependent nigrostriatal rescue along with increased high-affinity synaptosomal uptake of dopamine and counteraction of the motor deficits. The Nrf2/Wnt/β-catenin signaling is activated in the grafted astrocytes and upregulates their antioxidant self-defense mechanism, thereby restoring the impaired microenvironment*.* This switches the neurorescue-unfriendly environment of SN to a beneficial antioxidant/anti-inflammatory prosurvival milieu [170]. Similarly, engrafted type-2 astrocytes, a specific type of neonatal astrocytes with some characteristics of neural stem cells, have been used to repair the nigrostriatal pathway, improve symptoms, and induce functional reconstruction in 6-OHDA-induced PD rats [171]. Using single-cell RNA sequencing combined with comprehensive histological analyses, researchers have found that along with neurons, astrocytes are also a major component of both fetal and stem cell-derived intracerebral grafts after functional maturation, indicating a new source of functional astrocytes for transplantation [172]. Instead of focusing on single-function construction, transplantation of functional astrocytes would generate the entire range of protective functions of astrocytes, all of which are of great significance in inhibiting neuronal degeneration.

Astrocytes may also have the potential to convert to dopaminergic neurons. Human astrocytes in vitro have been reprogrammed into induced dopaminergic neurons with appropriate midbrain neuronal markers and electrical properties**.** In a 6-OHDA PD mouse model, dopaminergic neurons reprogrammed from adult striatal astrocytes are shown to be functional and can correct motor behavior, including gait impairments [173]. Recently, the conversion/cell reprogramming of astrocytes to neurons with dopaminergic features in the adult mouse striatum by CRISPR-CasRx-mediated knockdown of the RNA-binding protein PTB, known as PTBP1, has also been shown to alleviate motor deficits in 6-OHDA PD mice [174]. In a parallel approach, suppressing or deleting PTBP1 converts midbrain astrocytes to dopaminergic neurons in a 6-OHDA PD mouse model, which reconstructs the nigrostriatal circuit and restores the dopamine level [175]. Several mitochondrial proteins, including Sod1, Prdx2 and Arg2, are believed to participate in this process to improve the conversion efficiency [176]. These findings suggest that the in situ astrocyte-to-neuron conversion is a potentially powerful approach to treating neurodegeneration in PD.

## Conclusions

**Fig. 1:**
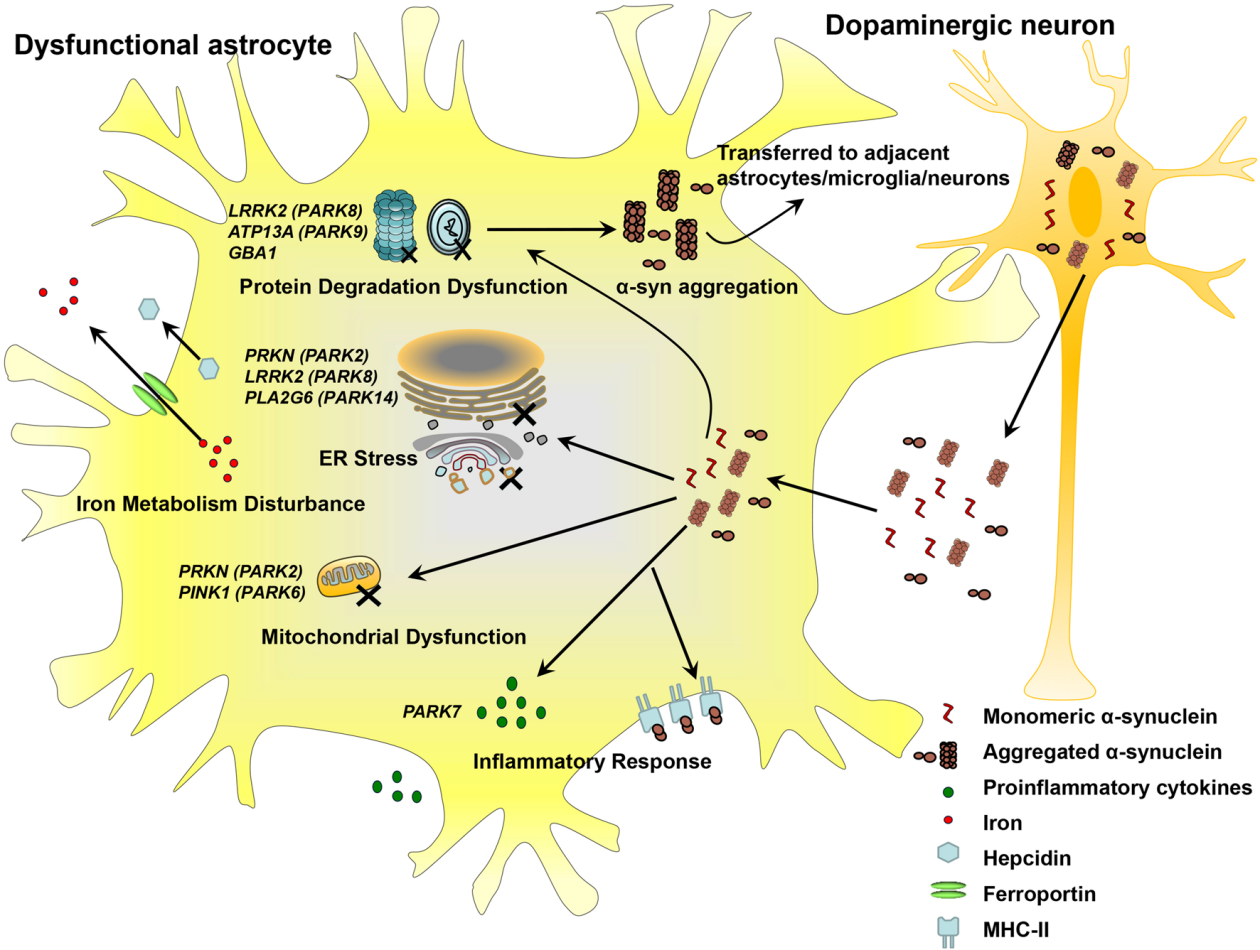
Astrocyte dysfunction induced by transmitted α-synuclein and PD-associated gene modulation. Astrocytes receive large amounts of α-synuclein released from dopaminergic neurons in the forms of monomeric and aggregated species. Both the proinflammatory phenotype and the reactive antigen (cross)-presenting phenotype of astrocytes are induced by transmitted α-synuclein. Astrocytes with *PARK7* mutations display a loss of anti-inflammatory function and enhanced proinflammatory responses. Transmitted α-synuclein impairs the astrocytic degradation capability and facilitates α-synuclein accumulation, and this can be aggravated by *LRRK2*, *GBA1* or *ATP13A2* mutations. Alpha-synuclein accumulation in astrocytes also induces mitochondrial dysfunction and endoplasmic reticulum stress. *PINK1* and *PRKN* mutations are involved in mitophagy deficiency and lead to dysfunctional mitochondria. Astrocytic ER stress might be a coordinator of the effects of *PRKN*, *LRRK2* and *PLA2G6* mutations. Iron transport in astrocytes, especially that via the iron exporter ferroportin and its ligand hepcidin, is pivotal for iron homeostasis in the surroundings. The pathological processes that occur in dysfunctional astrocytes, including protein degradation dysfunction, inflammatory response, ER stress, mitochondrial dysfunction, and iron metabolism disturbance, could be triggered by transmitted α-synuclein; moreover, the aggregated α-synuclein in astrocytes could be transferred to adjacent astrocytes, microglia and even neurons, thereby spreading the overall synucleinopathy and neurodegeneration

Glial cells in the brain are now being recognized as important contributors to or even as drivers of brain disease. In the diseased state of PD, astrocytes act as victims, accompanied by neurodegeneration. As ‘recipient’ cells of α-syn transferred from neurons, astrocytes experience an inflammatory response, dysfunction of protein degradation, mitochondrial dysfunction and ER stress induced by α-syn and thereby develop a dysfunctional phenotype that contributes to neurodegeneration. The mechanisms underlying the consequences of astrocytic alteration when exposed to transmitted α-syn have not been fully delineated. Astrocytes act not only as ‘recipient’ cells but also as secondary ‘donor’ cells to facilitate α-syn transmission, and the ways of implementing this process need to be explored further. Genes known to be causative in PD, such as *PARK7, GBA1, LRRK2, ATP13A2, PINK1*, *PRKN* and *PLA2G6,* are expressed in astrocytes and play important roles in astrocytic function. Mutations in these genes might trigger or exacerbate astrocytic dysfunction (Fig. 1). To clarify whether transferred α-syn together with PD-associated genetic mutations in astrocytes plays a profound role in astrocytic dysfunction and further neurodegeneration, more studies are needed. Whether astrocyte dysfunction initiates or merely exacerbates PD pathology also needs to be determined. With emerging evidence demonstrating a broader role of astrocyte dysfunction in PD pathogenesis, the maintenance of astrocytic function is becoming a target for rescuing neurodegeneration and improving the survival of neurons. Further investigation of how astrocytes contribute to PD will be significant for better understanding the disease and will have important implications for the development of new therapeutic approaches.

## Data Availability

Not applicable.

## Abbreviations

6-OHDA: 6-Hydroxydopamine
α-syn: Alpha-synuclein
BBB: Blood–brain barrier
CCL: C–C motif chemokine ligand
COX-2: Cyclooxygenase-2
Drd2: Dopamine D2 receptor
ER: Endoplasmic reticulum
ESC: Embryonic stem cell
FPN: Ferroportin
GCase: Glucocerebrosidase
GD: Gaucher disease
GFAP: Glial fibrillary acidic protein
HFE: Homeostatic iron regulator
IL: Interleukin
iPLA2: Ca^2^^+^-independent phospholipase A2
iPSC: Induced pluripotent stem cell
JAK1: Janus kinase 1
LAMP: Lysosomal-associated membrane protein
LPS: Lipopolysac-charide
LRRK: Leucine-rich repeat kinase 2
MHC: Major histocompatibility complex
MPTP: 1-Methyl 4-phenyl 1,2,3,6-tetrahydro pyridine
NF-κB: Nuclear factor kappa-B
NLRP3: Nod-like receptor protein 3
NOD2: Nucleotide-oligomerization domain receptor 2
Nrf2: Nuclear factor E2-related factor 2
Nurr1: Nuclear receptor related-1 protein
PD: Parkinson’s disease
PERK: PKR-like endoplasmic reticulum kinase
PFF: Preformed fibril
PINK1: PTEN -induced putative kinase 1
SNpc: Substantia nigra pars compacta
STAT1: Signal transducer and activator of transcription1
TLR: Toll-like receptor
TNT: Tunneling nanotube
UPR: Unfolded protein response
UPS: Ubiquitin–proteasome system

## References

[1] Poewe W, Seppi K, Tanner CM, Halliday GM, Brundin P, Volkmann J (2017). Parkinson disease. Nat Rev Dis Primers.

[2] Corti O, Lesage S, Brice A (2011). What genetics tells us about the causes and mechanisms of Parkinson's disease. Physiol Rev.

[3] Deng H, Wang P, Jankovic J (2018). The genetics of Parkinson disease. Ageing Res Rev.

[4] Kordower JH, Olanow CW, Dodiya HB, Chu Y, Beach TG, Adler CH (2013). Disease duration and the integrity of the nigrostriatal system in Parkinson's disease. Brain.

[5] Spillantini MG, Schmidt ML, Lee VM, Trojanowski JQ, Jakes R, Goedert M (1997). Alpha-synuclein in Lewy bodies. Nature.

[6] Goedert M (2015). NEURODEGENERATION. Alzheimer's and Parkinson's diseases: the prion concept in relation to assembled Aβ, tau, and α-synuclein. Science.

[7] Perez RG, Hastings TG (2004). Could a loss of alpha-synuclein function put dopaminergic neurons at risk?. J Neurochem.

[8] Auluck PK, Caraveo G, Lindquist S (2010). α-Synuclein: membrane interactions and toxicity in Parkinson's disease. Annu Rev Cell Dev Biol.

[9] Rekas A, Ahn KJ, Kim J, Carver JA (2012). The chaperone activity of α-synuclein: utilizing deletion mutants to map its interaction with target proteins. Proteins.

[10] Mor DE, Daniels MJ, Ischiropoulos H (2019). The usual suspects, dopamine and alpha-synuclein, conspire to cause neurodegeneration. Mov Disord.

[11] Gu XL, Long CX, Sun L, Xie C, Lin X, Cai H (2010). Astrocytic expression of Parkinson's disease-related A53T alpha-synuclein causes neurodegeneration in mice. Mol Brain.

[12] Sonninen TM, Hämäläinen RH (2020). Metabolic alterations in Parkinson's disease astrocytes. Sci Rep.

[13] Lee HJ, Suk JE, Patrick C, Bae EJ, Cho JH, Rho S (2010). Direct transfer of alpha-synuclein from neuron to astroglia causes inflammatory responses in synucleinopathies. J Biol Chem.

[14] Chavarría C, Rodríguez-Bottero S, Quijano C, Cassina P, Souza JM (2018). Impact of monomeric, oligomeric and fibrillar alpha-synuclein on astrocyte reactivity and toxicity to neurons. Biochem J.

[15] Brundin P, Melki R (2017). Prying into the prion hypothesis for Parkinson's disease. J Neurosci.

[16] Cavaliere F, Cerf L, Dehay B, Ramos-Gonzalez P, De Giorgi F, Bourdenx M (2017). In vitro α-synuclein neurotoxicity and spreading among neurons and astrocytes using Lewy body extracts from Parkinson disease brains. Neurobiol Dis.

[17] Braidy N, Gai WP, Xu YH, Sachdev P, Guillemin GJ, Jiang XM (2013). Uptake and mitochondrial dysfunction of alpha-synuclein in human astrocytes, cortical neurons and fibroblasts. Transl Neurodegener.

[18] Lindström V, Gustafsson G, Sanders LH, Howlett EH, Sigvardson J, Kasrayan A (2017). Extensive uptake of α-synuclein oligomers in astrocytes results in sustained intracellular deposits and mitochondrial damage. Mol Cell Neurosci.

[19] Filippini A, Mutti V, Faustini G, Longhena F, Ramazzina I, Rizzi F (2021). Extracellular clusterin limits the uptake of α-synuclein fibrils by murine and human astrocytes. Glia.

[20] Rostami J, Holmqvist S (2017). Human astrocytes transfer aggregated alpha-synuclein via tunneling nanotubes. J Neurosci.

[21] Verkhratsky A, Matteoli M, Parpura V, Mothet JP, Zorec R (2016). Astrocytes as secretory cells of the central nervous system: idiosyncrasies of vesicular secretion. EMBO J.

[22] Delenclos M, Trendafilova T, Mahesh D, Baine AM, Moussaud S, Yan IK (2017). Investigation of endocytic pathways for the internalization of exosome-associated oligomeric alpha-synuclein. Front Neurosci.

[23] Meng Y, Ding J, Li C, Fan H, He Y, Qiu P (2020). Transfer of pathological α-synuclein from neurons to astrocytes via exosomes causes inflammatory responses after METH exposure. Toxicol Lett.

[24] Alexei Verkhratsky AB (2013). Numbers: how many glial cells are in the brain? In glial physiology and pathophysiology.

[25] Sofroniew MV, Vinters HV (2010). Astrocytes: biology and pathology. Acta Neuropathol.

[26] Khakh BS, Sofroniew MV (2015). Diversity of astrocyte functions and phenotypes in neural circuits. Nat Neurosci.

[27] Lee JH, Kim JY, Noh S, Lee H, Lee SY, Mun JY (2021). Astrocytes phagocytose adult hippocampal synapses for circuit homeostasis. Nature.

[28] Takano T, Wallace JT, Baldwin KT, Purkey AM, Uezu A, Courtland JL (2020). Chemico-genetic discovery of astrocytic control of inhibition in vivo. Nature.

[29] Pannasch U, Freche D, Dallérac G, Ghézali G, Escartin C, Ezan P (2014). Connexin 30 sets synaptic strength by controlling astroglial synapse invasion. Nat Neurosci.

[30] Liddelow SA, Guttenplan KA, Clarke LE, Bennett FC, Bohlen CJ, Schirmer L (2017). Neurotoxic reactive astrocytes are induced by activated microglia. Nature.

[31] Ransohoff RM (2016). How neuroinflammation contributes to neurodegeneration. Science.

[32] Yang L, Mao K, Yu H, Chen J (2020). Neuroinflammatory responses and Parkinson' disease: pathogenic mechanisms and therapeutic targets. J Neuroimmune Pharmacol.

[33] Li T, Le W (2020). Biomarkers for Parkinson's disease: how good are they?. Neurosci Bull.

[34] Joshi AU, Minhas PS, Liddelow SA (2019). Fragmented mitochondria released from microglia trigger A1 astrocytic response and propagate inflammatory neurodegeneration. Nat Neurosci.

[35] Wang Q, Liu Y, Zhou J (2015). Neuroinflammation in Parkinson's disease and its potential as therapeutic target. Transl Neurodegener.

[36] Yun SP, Kam TI, Panicker N, Kim S, Oh Y, Park JS (2018). Block of A1 astrocyte conversion by microglia is neuroprotective in models of Parkinson's disease. Nat Med.

[37] Voet S, Srinivasan S, Lamkanfi M, van Loo G (2019). Inflammasomes in neuroinflammatory and neurodegenerative diseases. EMBO Mol Med.

[38] Zhu J, Sun T, Zhang J, Liu Y, Wang D, Zhu H (2020). Drd2 biased agonist prevents neurodegeneration against NLRP3 inflammasome in Parkinson's disease model via a β-arrestin2-biased mechanism. Brain Behav Immun.

[39] Zhou Y, Lu M, Du RH, Qiao C, Jiang CY, Zhang KZ (2016). MicroRNA-7 targets Nod-like receptor protein 3 inflammasome to modulate neuroinflammation in the pathogenesis of Parkinson's disease. Mol Neurodegener.

[40] Linnerbauer M, Wheeler MA, Quintana FJ (2020). Astrocyte Crosstalk in CNS Inflammation. Neuron.

[41] Rannikko EH, Weber SS, Kahle PJ (2015). Exogenous α-synuclein induces toll-like receptor 4 dependent inflammatory responses in astrocytes. BMC Neurosci.

[42] Aflaki E, Stubblefield BK, McGlinchey RP, McMahon B, Ory DS, Sidransky E (2020). A characterization of Gaucher iPS-derived astrocytes: potential implications for Parkinson's disease. Neurobiol Dis.

[43] Fellner L, Irschick R, Schanda K, Reindl M, Klimaschewski L, Poewe W (2013). Toll-like receptor 4 is required for α-synuclein dependent activation of microglia and astroglia. Glia.

[44] Kim C, Spencer B, Rockenstein E, Yamakado H, Mante M, Adame A (2018). Immunotherapy targeting toll-like receptor 2 alleviates neurodegeneration in models of synucleinopathy by modulating α-synuclein transmission and neuroinflammation. Mol Neurodegener.

[45] Kim C, Kwon S, Iba M, Spencer B, Rockenstein E, Mante M (2021). Effects of innate immune receptor stimulation on extracellular α-synuclein uptake and degradation by brain resident cells. Exp Mol Med.

[46] Hughes CD, Choi ML, Ryten M, Hopkins L, Drews A, Botía JA (2019). Picomolar concentrations of oligomeric alpha-synuclein sensitizes TLR4 to play an initiating role in Parkinson's disease pathogenesis. Acta Neuropathol.

[47] Saijo K, Winner B, Carson CT, Collier JG, Boyer L, Rosenfeld MG (2009). A Nurr1/CoREST pathway in microglia and astrocytes protects dopaminergic neurons from inflammation-induced death. Cell.

[48] Popichak KA, Hammond SL, Moreno JA, Afzali MF, Backos DS, Slayden RD (2018). Compensatory expression of Nur77 and Nurr1 regulates NF-κB-dependent inflammatory signaling in astrocytes. Mol Pharmacol.

[49] Shao W, Zhang SZ, Tang M, Zhang XH, Zhou Z, Yin YQ (2013). Suppression of neuroinflammation by astrocytic dopamine D2 receptors via αB-crystallin. Nature.

[50] Zhu J, Hu Z, Han X, Wang D, Jiang Q, Ding J (2018). Dopamine D2 receptor restricts astrocytic NLRP3 inflammasome activation via enhancing the interaction of β-arrestin2 and NLRP3. Cell Death Differ.

[51] Du RH, Zhou Y, Xia ML, Lu M, Ding JH, Hu G (2018). α-Synuclein disrupts the anti-inflammatory role of Drd2 via interfering β-arrestin2-TAB1 interaction in astrocytes. J Neuroinflamm.

[52] Russ K, Teku G, Bousset L, Redeker V, Piel S, Savchenko E (2021). TNF-α and α-synuclein fibrils differently regulate human astrocyte immune reactivity and impair mitochondrial respiration. Cell Rep.

[53] Rostami J, Fotaki G, Sirois J, Mzezewa R, Bergström J, Essand M (2020). Astrocytes have the capacity to act as antigen-presenting cells in the Parkinson's disease brain. J Neuroinflamm.

[54] Dolgacheva LP, Berezhnov AV, Fedotova EI, Zinchenko VP, Abramov AY (2019). Role of DJ-1 in the mechanism of pathogenesis of Parkinson's disease. J Bioenerg Biomembr.

[55] Bandopadhyay R, Kingsbury AE, Cookson MR, Reid AR, Evans IM, Hope AD (2004). The expression of DJ-1 (PARK7) in normal human CNS and idiopathic Parkinson's disease. Brain.

[56] Waak J, Weber SS, Waldenmaier A, Görner K, Alunni-Fabbroni M, Schell H (2009). Regulation of astrocyte inflammatory responses by the Parkinson's disease-associated gene DJ-1. FASEB J.

[57] Kim JH, Choi DJ, Jeong HK, Kim J, Kim DW, Choi SY (2013). DJ-1 facilitates the interaction between STAT1 and its phosphatase, SHP-1, in brain microglia and astrocytes: a novel anti-inflammatory function of DJ-1. Neurobiol Dis.

[58] Kim JH, Jou I, Joe EH (2014). Suppression of miR-155 expression in IFN-γ-treated astrocytes and microglia by DJ-1: a possible mechanism for maintaining SOCS1 expression. Exp Neurobiol.

[59] Choi DJ, An J, Jou I, Park SM, Joe EH (2019). A Parkinson's disease gene, DJ-1, regulates anti-inflammatory roles of astrocytes through prostaglandin D(2) synthase expression. Neurobiol Dis.

[60] Choi DJ, Eun JH, Kim BG, Jou I, Park SM, Joe EH (2018). A Parkinson's disease gene, DJ-1, repairs brain injury through Sox9 stabilization and astrogliosis. Glia.

[61] Choi DJ, Yang H, Gaire S, Lee KA, An J, Kim BG (2020). Critical roles of astrocytic-CCL2-dependent monocyte infiltration in a DJ-1 knockout mouse model of delayed brain repair. Glia.

[62] Frøyset AK, Edson AJ, Gharbi N, Khan EA, Dondorp D, Bai Q (2018). Astroglial DJ-1 over-expression up-regulates proteins involved in redox regulation and is neuroprotective in vivo. Redox Biol.

[63] De Miranda BR, Rocha EM, Bai Q, El Ayadi A, Hinkle D, Burton EA (2018). Astrocyte-specific DJ-1 overexpression protects against rotenone-induced neurotoxicity in a rat model of Parkinson's disease. Neurobiol Dis.

[64] Ebrahimi-Fakhari D, Wahlster L, McLean PJ (2012). Protein degradation pathways in Parkinson's disease: curse or blessing. Acta Neuropathol.

[65] Galves M, Rathi R, Prag G, Ashkenazi A (2019). Ubiquitin signaling and degradation of aggregate-prone proteins. Trends Biochem Sci.

[66] Loria F, Vargas JY, Bousset L, Syan S, Salles A, Melki R (2017). α-Synuclein transfer between neurons and astrocytes indicates that astrocytes play a role in degradation rather than in spreading. Acta Neuropathol.

[67] Tsunemi T, Ishiguro Y, Yoroisaka A, Valdez C, Miyamoto K, Ishikawa K (2020). Astrocytes protect human dopaminergic neurons from α-synuclein accumulation and propagation. J Neurosci.

[68] Hua J, Yin N, Xu S, Chen Q, Tao T, Zhang J (2019). Enhancing the astrocytic clearance of extracellular α-synuclein aggregates by Ginkgolides attenuates neural cell injury. Cell Mol Neurobiol.

[69] Choi I, Zhang Y, Seegobin SP (2020). Microglia clear neuron-released α-synuclein via selective autophagy and prevent neurodegeneration. Nat Commun.

[70] Xia Y, Zhang G, Han C, Ma K, Guo X, Wan F (2019). Microglia as modulators of exosomal alpha-synuclein transmission. Cell Death Dis.

[71] Guo M, Wang J, Zhao Y, Feng Y, Han S, Dong Q (2020). Microglial exosomes facilitate α-synuclein transmission in Parkinson's disease. Brain.

[72] Rostami J, Mothes T, Kolahdouzan M, Eriksson O, Moslem M, Bergström J (2021). Crosstalk between astrocytes and microglia results in increased degradation of α-synuclein and amyloid-β aggregates. J Neuroinflamm.

[73] Sacino AN, Brooks M, McKinney AB, Thomas MA, Shaw G, Golde TE (2014). Brain injection of α-synuclein induces multiple proteinopathies, gliosis, and a neuronal injury marker. J Neurosci.

[74] Rutherford NJ, Sacino AN, Brooks M, Ceballos-Diaz C, Ladd TB, Howard JK (2015). Studies of lipopolysaccharide effects on the induction of α-synuclein pathology by exogenous fibrils in transgenic mice. Mol Neurodegener.

[75] Lu SZ, Guo YS, Liang PZ, Zhang SZ, Yin S, Yin YQ (2019). Suppression of astrocytic autophagy by αB-crystallin contributes to α-synuclein inclusion formation. Transl Neurodegener.

[76] Liu Y, Zhou Q, Tang M, Fu N, Shao W, Zhang S (2015). Upregulation of alphaB-crystallin expression in the substantia nigra of patients with Parkinson's disease. Neurobiol Aging.

[77] Wakabayashi K, Hayashi S, Yoshimoto M, Kudo H, Takahashi H (2000). NACP/alpha-synuclein-positive filamentous inclusions in astrocytes and oligodendrocytes of Parkinson's disease brains. Acta Neuropathol.

[78] Braak H, Sastre M, Del Tredici K (2007). Development of alpha-synuclein immunoreactive astrocytes in the forebrain parallels stages of intraneuronal pathology in sporadic Parkinson's disease. Acta Neuropathol.

[79] Stevenson TJ, Murray HC, Turner C, Faull RLM, Dieriks BV, Curtis MA (2020). α-synuclein inclusions are abundant in non-neuronal cells in the anterior olfactory nucleus of the Parkinson's disease olfactory bulb. Sci Rep.

[80] Zimprich A, Biskup S, Leitner P, Lichtner P, Farrer M, Lincoln S (2004). Mutations in LRRK2 cause autosomal-dominant parkinsonism with pleomorphic pathology. Neuron.

[81] Greggio E, Jain S, Kingsbury A, Bandopadhyay R, Lewis P, Kaganovich A (2006). Kinase activity is required for the toxic effects of mutant LRRK2/dardarin. Neurobiol Dis.

[82] Li JQ, Tan L, Yu JT (2014). The role of the LRRK2 gene in Parkinsonism. Mol Neurodegener.

[83] Di Maio R, Hoffman EK, Rocha EM, Keeney MT, De Miranda BR, Sanders LH (2018). LRRK2 activation in idiopathic Parkinson's disease. Sci Transl Med..

[84] Miklossy J, Arai T, Guo JP, Klegeris A, Yu S, McGeer EG (2006). LRRK2 expression in normal and pathologic human brain and in human cell lines. J Neuropathol Exp Neurol.

[85] Henry AG, Aghamohammadzadeh S, Samaroo H, Chen Y, Mou K, Needle E (2015). Pathogenic LRRK2 mutations, through increased kinase activity, produce enlarged lysosomes with reduced degradative capacity and increase ATP13A2 expression. Hum Mol Genet.

[86] di Domenico A, Carola G, Calatayud C, Pons-Espinal M, Muñoz JP, Richaud-Patin Y (2019). Patient-specific iPSC-derived astrocytes contribute to non-cell-autonomous neurodegeneration in Parkinson's disease. Stem Cell Rep.

[87] Oh SH, Kim HN, Park HJ, Shin JY, Kim DY, Lee PH (2017). The Cleavage effect of mesenchymal stem cell and its derived matrix metalloproteinase-2 on extracellular α-synuclein aggregates in parkinsonian models. Stem Cells Transl Med.

[88] Booth HDE, Wessely F, Connor-Robson N, Rinaldi F, Vowles J, Browne C (2019). RNA sequencing reveals MMP2 and TGFB1 downregulation in LRRK2 G2019S Parkinson's iPSC-derived astrocytes. Neurobiol Dis.

[89] Streubel-Gallasch L, Giusti V, Sandre M, Tessari I, Plotegher N, Giusto E (2021). Parkinson's disease-associated LRRK2 interferes with astrocyte-mediated alpha-synuclein clearance. Mol Neurobiol.

[90] Sidransky E, Nalls MA, Aasly JO, Aharon-Peretz J, Annesi G, Barbosa ER (2009). Multicenter analysis of glucocerebrosidase mutations in Parkinson's disease. N Engl J Med.

[91] Aflaki E, Westbroek W, Sidransky E (2017). The complicated relationship between Gaucher disease and Parkinsonism: insights from a rare disease. Neuron.

[92] Mazzulli JR, Xu YH, Sun Y, Knight AL, McLean PJ, Caldwell GA (2011). Gaucher disease glucocerebrosidase and α-synuclein form a bidirectional pathogenic loop in synucleinopathies. Cell.

[93] Schöndorf DC, Aureli M, McAllister FE, Hindley CJ, Mayer F, Schmid B (2014). iPSC-derived neurons from GBA1-associated Parkinson's disease patients show autophagic defects and impaired calcium homeostasis. Nat Commun.

[94] Do J, McKinney C, Sharma P, Sidransky E (2019). Glucocerebrosidase and its relevance to Parkinson disease. Mol Neurodegener.

[95] Ginns EI, Mak SK, Ko N, Karlgren J, Akbarian S, Chou VP (2014). Neuroinflammation and α-synuclein accumulation in response to glucocerebrosidase deficiency are accompanied by synaptic dysfunction. Mol Genet Metab.

[96] Osellame LD, Rahim AA, Hargreaves IP, Gegg ME, Richard-Londt A, Brandner S (2013). Mitochondria and quality control defects in a mouse model of Gaucher disease–links to Parkinson's disease. Cell Metab.

[97] Henderson MX, Sedor S, McGeary I, Cornblath EJ, Peng C, Riddle DM (2020). Glucocerebrosidase activity modulates neuronal susceptibility to pathological α-synuclein insult. Neuron.

[98] Sanyal A, DeAndrade MP, Novis HS, Lin S, Chang J, Lengacher N (2020). Lysosome and inflammatory defects in GBA1-mutant astrocytes are normalized by LRRK2 inhibition. Mov Disord.

[99] Soria FN, Engeln M, Martinez-Vicente M, Glangetas C, López-González MJ, Dovero S (2017). Glucocerebrosidase deficiency in dopaminergic neurons induces microglial activation without neurodegeneration. Hum Mol Genet.

[100] Ramirez A, Heimbach A, Gründemann J, Stiller B, Hampshire D, Cid LP (2006). Hereditary parkinsonism with dementia is caused by mutations in ATP13A2, encoding a lysosomal type 5 P-type ATPase. Nat Genet.

[101] Rochet JC (2012). New insights into lysosomal dysfunction in Parkinson’s disease: an emerging role for ATP13A2. Mov Disord.

[102] Park JS, Blair NF, Sue CM (2015). The role of ATP13A2 in Parkinson's disease: clinical phenotypes and molecular mechanisms. Mov Disord.

[103] Baesler J, Kopp JF, Pohl G, Aschner M, Haase H, Schwerdtle T (2019). Zn homeostasis in genetic models of Parkinson's disease in Caenorhabditis elegans. J Trace Elem Med Biol.

[104] Qiao C, Yin N, Gu HY, Zhu JL, Ding JH, Lu M (2016). Atp13a2 deficiency aggravates astrocyte-mediated neuroinflammation via NLRP3 inflammasome activation. CNS Neurosci Ther.

[105] Zhang Y, Chen K, Sloan SA, Bennett ML, Scholze AR, O'Keeffe S (2014). An RNA-sequencing transcriptome and splicing database of glia, neurons, and vascular cells of the cerebral cortex. J Neurosci.

[106] Gollihue JL, Norris CM (2020). Astrocyte mitochondria: central players and potential therapeutic targets for neurodegenerative diseases and injury. Ageing Res Rev.

[107] Gustafsson G, Lindström V, Rostami J, Nordström E, Lannfelt L, Bergström J (2017). Alpha-synuclein oligomer-selective antibodies reduce intracellular accumulation and mitochondrial impairment in alpha-synuclein exposed astrocytes. J Neuroinflamm.

[108] Schmidt S, Linnartz B, Mendritzki S, Sczepan T, Lübbert M, Stichel CC (2011). Genetic mouse models for Parkinson's disease display severe pathology in glial cell mitochondria. Hum Mol Genet.

[109] Erustes AG, Stefani FY, Terashima JY, Stilhano RS, Monteforte PT, da Silva Pereira GJ (2018). Overexpression of α-synuclein in an astrocyte cell line promotes autophagy inhibition and apoptosis. J Neurosci Res.

[110] Falchi AM, Sogos V, Saba F, Piras M, Congiu T, Piludu M (2013). Astrocytes shed large membrane vesicles that contain mitochondria, lipid droplets and ATP. Histochem Cell Biol.

[111] Hayakawa K, Esposito E, Wang X, Terasaki Y, Liu Y, Xing C (2016). Transfer of mitochondria from astrocytes to neurons after stroke. Nature.

[112] Kuter KZ, Olech Ł, Dencher NA (2019). Increased energetic demand supported by mitochondrial electron transfer chain and astrocyte assistance is essential to maintain the compensatory ability of the dopaminergic neurons in an animal model of early Parkinson's disease. Mitochondrion.

[113] Cheng XY, Biswas S, Li J, Mao CJ, Chechneva O, Chen J (2020). Human iPSCs derived astrocytes rescue rotenone-induced mitochondrial dysfunction and dopaminergic neurodegeneration in vitro by donating functional mitochondria. Transl Neurodegener.

[114] Von Stockum S, Nardin A, Schrepfer E, Ziviani E (2016). Mitochondrial dynamics and mitophagy in Parkinson's disease: a fly point of view. Neurobiol Dis.

[115] Hu ZL, Sun T, Lu M, Ding JH, Du RH, Hu G (2019). Kir6.1/K-ATP channel on astrocytes protects against dopaminergic neurodegeneration in the MPTP mouse model of Parkinson's disease via promoting mitophagy. Brain Behav Immun.

[116] Thomzig A, Wenzel M, Karschin C, Eaton MJ, Skatchkov SN, Karschin A (2001). Kir61 is the principal pore-forming subunit of astrocyte but not neuronal plasma membrane K-ATP channels. Mol Cell Neurosci.

[117] Tinker A, Aziz Q, Li Y, Specterman M (2018). ATP-sensitive potassium channels and their physiological and pathophysiological roles. Compr Physiol.

[118] Morales I, Sanchez A, Puertas-Avendaño R, Rodriguez-Sabate C, Perez-Barreto A, Rodriguez M (2020). Neuroglial transmitophagy and Parkinson's disease. Glia.

[119] Morales I, Puertas-Avendaño R, Sanchez A, Perez-Barreto A, Rodriguez-Sabate C, Rodriguez M (2021). Astrocytes and retrograde degeneration of nigrostriatal dopaminergic neurons in parkinson’s disease: removing axonal debris. Transl Neurodegener.

[120] Narendra DP, Jin SM, Tanaka A, Suen DF, Gautier CA, Shen J (2010). PINK1 is selectively stabilized on impaired mitochondria to activate Parkin. PLoS Biol.

[121] Okatsu K, Oka T, Iguchi M, Imamura K, Kosako H, Tani N (2012). PINK1 autophosphorylation upon membrane potential dissipation is essential for Parkin recruitment to damaged mitochondria. Nat Commun.

[122] Kano M, Takanashi M (2020). Reduced astrocytic reactivity in human brains and midbrain organoids with PRKN mutations. NPJ Parkinsons Dis.

[123] Choi I, Kim J, Jeong HK, Kim B, Jou I, Park SM (2013). PINK1 deficiency attenuates astrocyte proliferation through mitochondrial dysfunction, reduced AKT and increased p38 MAPK activation, and downregulation of EGFR. Glia.

[124] Choi I, Choi DJ, Yang H, Woo JH, Chang MY, Kim JY (2016). PINK1 expression increases during brain development and stem cell differentiation, and affects the development of GFAP-positive astrocytes. Mol Brain.

[125] Martin-Jiménez CA, García-Vega Á, Cabezas R, Aliev G, Echeverria V, González J (2017). Astrocytes and endoplasmic reticulum stress: a bridge between obesity and neurodegenerative diseases. Prog Neurobiol.

[126] Walter P, Ron D (2011). The unfolded protein response: from stress pathway to homeostatic regulation. Science.

[127] Mercado G, Valdés P, Hetz C (2013). An ERcentric view of Parkinson's disease. Trends Mol Med.

[128] Colla E (2019). Linking the endoplasmic reticulum to Parkinson's disease and alpha-synucleinopathy. Front Neurosci.

[129] Jiang P, Gan M, Ebrahim AS, Lin WL, Melrose HL, Yen SH (2010). ER stress response plays an important role in aggregation of α-synuclein. Mol Neurodegener.

[130] Castillo-Carranza DL, Zhang Y, Guerrero-Muñoz MJ, Kayed R, Rincon-Limas DE, Fernandez-Funez P (2012). Differential activation of the ER stress factor XBP1 by oligomeric assemblies. Neurochem Res.

[131] Mi X, Li Q, Wen X, Xie J, Wang Y, Song N (2021). Extracellular α-synuclein modulates iron metabolism related proteins via endoplasmic reticulum stress in MES23.5 dopaminergic cells. Neurochem Res.

[132] Liu M, Qin L, Wang L, Tan J, Zhang H, Tang J (2018). α-synuclein induces apoptosis of astrocytes by causing dysfunction of the endoplasmic reticulum-Golgi compartment. Mol Med Rep.

[133] Lee JH, Han JH, Kim H, Park SM, Joe EH, Jou I (2019). Parkinson's disease-associated LRRK2-G2019S mutant acts through regulation of SERCA activity to control ER stress in astrocytes. Acta Neuropathol Commun.

[134] Meares GP, Liu Y, Rajbhandari R, Qin H, Nozell SE, Mobley JA (2014). PERK-dependent activation of JAK1 and STAT3 contributes to endoplasmic reticulum stress-induced inflammation. Mol Cell Biol.

[135] Sanchez CL, Sims SG, Nowery JD, Meares GP (2019). Endoplasmic reticulum stress differentially modulates the IL-6 family of cytokines in murine astrocytes and macrophages. Sci Rep.

[136] Guthrie LN, Abiraman K, Plyler ES, Sprenkle NT, Gibson SA, McFarland BC (2016). Attenuation of PKR-like ER kinase (PERK) signaling selectively controls endoplasmic reticulum stress-induced inflammation without compromising immunological responses. J Biol Chem.

[137] Smith HL, Freeman OJ, Butcher AJ, Holmqvist S, Humoud I, Schätzl T (2020). Astrocyte unfolded protein response induces a specific reactivity state that causes non-cell-autonomous neuronal degeneration. Neuron.

[138] Wang Y, Chen Y, Zhou Q, Xu J, Qian Q, Ni P (2018). Mild endoplasmic reticulum stress protects against lipopolysaccharide-induced astrocytic activation and blood-brain barrier hyperpermeability. Front Cell Neurosci.

[139] Sprenkle NT, Lahiri A, Simpkins JW, Meares GP (2019). Endoplasmic reticulum stress is transmissible in vitro between cells of the central nervous system. J Neurochem.

[140] van Ziel AM, Wolzak K, Nölle A, Hoetjes PJ, Berenjeno-Correa E, van Anken E (2020). No evidence for cell-to-cell transmission of the unfolded protein response in cell culture. J Neurochem.

[141] Bignon Y, Poindessous V, Rampoldi L, Haldys V, Pallet N (2020). Chemically based transmissible ER stress protocols are unsuitable to study cell-to-cell UPR transmission. Biochem J.

[142] Ramonet D, Daher JP, Lin BM, Stafa K, Kim J, Banerjee R (2011). Dopaminergic neuronal loss, reduced neurite complexity and autophagic abnormalities in transgenic mice expressing G2019S mutant LRRK2. PLoS One.

[143] Yuan Y, Cao P, Smith MA, Kramp K, Huang Y, Hisamoto N (2011). Dysregulated LRRK2 signaling in response to endoplasmic reticulum stress leads to dopaminergic neuron degeneration in C. elegans. PLoS One.

[144] Ledesma MD, Galvan C, Hellias B, Dotti C, Jensen PH (2002). Astrocytic but not neuronal increased expression and redistribution of parkin during unfolded protein stress. J Neurochem.

[145] Keestra-Gounder AM, Byndloss MX, Seyffert N, Young BM, Chávez-Arroyo A, Tsai AY (2016). NOD1 and NOD2 signalling links ER stress with inflammation. Nature.

[146] Singh K, Han K, Tilve S, Wu K, Geller HM, Sack MN (2018). Parkin targets NOD2 to regulate astrocyte endoplasmic reticulum stress and inflammation. Glia.

[147] Shi CH, Tang BS, Wang L, Lv ZY, Wang J, Luo LZ (2011). PLA2G6 gene mutation in autosomal recessive early-onset parkinsonism in a Chinese cohort. Neurology.

[148] Trinh J, Lohmann K, Baumann H, Balck A, Borsche M, Brüggemann N (2019). Utility and implications of exome sequencing in early-onset Parkinson's disease. Mov Disord.

[149] Chiu CC, Lu CS, Weng YH, Chen YL, Huang YZ, Chen RS (2019). PARK14 (D331Y) PLA2G6 causes early-onset degeneration of Substantia Nigra dopaminergic neurons by inducing mitochondrial dysfunction, ER stress, mitophagy impairment and transcriptional dysregulation in a Knockin mouse model. Mol Neurobiol.

[150] Ke M, Chong CM, Zeng H, Huang M, Huang Z, Zhang K (2020). Azoramide protects iPSC-derived dopaminergic neurons with PLA2G6 D331Y mutation through restoring ER function and CREB signaling. Cell Death Dis.

[151] Mori A, Hatano T, Inoshita T, Shiba-Fukushima K, Koinuma T, Meng H (2019). Parkinson's disease-associated iPLA2-VIA/PLA2G6 regulates neuronal functions and α-synuclein stability through membrane remodeling. Proc Natl Acad Sci U S A.

[152] Ward RJ, Zucca FA, Duyn JH, Crichton RR, Zecca L (2014). The role of iron in brain ageing and neurodegenerative disorders. Lancet Neurol.

[153] Healy S, McMahon JM, FitzGerald U (2017). Modelling iron mismanagement in neurodegenerative disease in vitro: paradigms, pitfalls, possibilities & practical considerations. Prog Neurobiol.

[154] Yanase K, Uemura N, Chiba Y, Murakami R, Fujihara R, Matsumoto K (2020). Immunoreactivities for hepcidin, ferroportin, and hephaestin in astrocytes and choroid plexus epithelium of human brains. Neuropathology.

[155] Ashraf A, Michaelides C, Walker TA, Ekonomou A, Suessmilch M, Sriskanthanathan A (2019). Regional distributions of iron, copper and zinc and their relationships with glia in a normal aging mouse model. Front Aging Neurosci.

[156] Song N, Wang J, Jiang H, Xie J (2018). Astroglial and microglial contributions to iron metabolism disturbance in Parkinson's disease. Biochim Biophys Acta Mol Basis Dis.

[157] Cui J, Guo X, Li Q, Song N, Xie J (2020). Hepcidin-to-ferritin ratio is decreased in astrocytes with extracellular alpha-synuclein and iron exposure. Front Cell Neurosci.

[158] McCarthy RC, Kosman DJ (2014). Glial cell ceruloplasmin and hepcidin differentially regulate iron efflux from brain microvascular endothelial cells. PLoS One.

[159] Raha-Chowdhury R, Raha AA, Forostyak S, Zhao JW, Stott SR, Bomford A (2015). Expression and cellular localization of hepcidin mRNA and protein in normal rat brain. BMC Neurosci.

[160] Xu Y, Zhang Y, Zhang JH, Han K, Zhang X, Bai X (2020). Astrocyte hepcidin ameliorates neuronal loss through attenuating brain iron deposition and oxidative stress in APP/PS1 mice. Free Radic Biol Med.

[161] Liang T, Qian ZM, Mu MD, Yung WH, Ke Y (2020). Brain hepcidin suppresses major pathologies in experimental parkinsonism. iScience..

[162] Cerri S, Mus L, Blandini F (2019). Parkinson's disease in women and men: what's the difference?. J Parkinsons Dis.

[163] Xu M, Tan X, Li N, Wu H, Wang Y, Xie J (2019). Differential regulation of estrogen in iron metabolism in astrocytes and neurons. J Cell Physiol.

[164] Nixon AM, Meadowcroft MD, Neely EB, Snyder AM, Purnell CJ, Wright J (2018). HFE genotype restricts the response to paraquat in a mouse model of neurotoxicity. J Neurochem.

[165] Kim Y, Connor JR (2020). The roles of iron and HFE genotype in neurological diseases. Mol Asp Med.

[166] Song IY, Snyder AM, Kim Y, Neely EB, Wade QW, Connor JR (2020). The Nrf2-mediated defense mechanism associated with HFE genotype limits vulnerability to oxidative stress-induced toxicity. Toxicology.

[167] Thomas GEC, Zarkali A, Ryten M, Shmueli K, Gil-Martinez AL, Leyland LA (2021). Regional brain iron and gene expression provide insights into neurodegeneration in Parkinson's disease. Brain.

[168] Kuter K, Olech Ł, Głowacka U, Paleczna M (2019). Astrocyte support is important for the compensatory potential of the nigrostriatal system neurons during early neurodegeneration. J Neurochem.

[169] Song JJ, Oh SM, Kwon OC, Wulansari N, Lee HS, Chang MY (2018). Cografting astrocytes improves cell therapeutic outcomes in a Parkinson's disease model. J Clin Investig.

[170] Serapide MF, L'Episcopo F, Tirolo C, Testa N, Caniglia S, Giachino C (2020). Boosting antioxidant self-defenses by grafting astrocytes rejuvenates the aged microenvironment and mitigates nigrostriatal toxicity in Parkinsonian brain via an Nrf2-driven Wnt/β-catenin prosurvival axis. Front Aging Neurosci.

[171] Sun Y, Lu XJ, Fu X, Zhang Y, Zhan Y, Liu J (2021). Engrafted primary type-2 astrocytes improve the recovery of the nigrostriatal pathway in a rat model of Parkinson's disease. Mol Cell Biochem.

[172] Tiklová K, Nolbrant S (2020). Single cell transcriptomics identifies stem cell-derived graft composition in a model of Parkinson's disease. Nat Commun.

[173] Rivetti di Val Cervo P, Romanov RA, Spigolon G, Masini D, Martín-Montañez E, Toledo EM (2017). Induction of functional dopamine neurons from human astrocytes in vitro and mouse astrocytes in a Parkinson's disease model. Nat Biotechnol.

[174] Zhou H, Su J, Hu X, Zhou C, Li H, Chen Z (2020). Glia-to-neuron conversion by CRISPR-CasRx alleviates symptoms of neurological disease in mice. Cell.

[175] Qian H, Kang X, Hu J, Zhang D (2020). Reversing a model of Parkinson's disease with in situ converted nigral neurons. Nature.

[176] Russo GL, Sonsalla G, Natarajan P, Breunig CT, Bulli G, Merl-Pham J (2021). CRISPR-mediated induction of neuron-enriched mitochondrial proteins boosts direct glia-to-neuron conversion. Cell Stem Cell.

